# Functional Analysis of *GhRNG1L* Reveals Its Positive Role in Drought Tolerance Mediated by *GhDREB2B* in Cotton

**DOI:** 10.3390/plants15172636

**Published:** 2026-08-28

**Authors:** Mengyan Wang, Li An, Xiaokang Fu, Chao Shen, Jianhua Lu, Hongmei Wu, Yanna Gao, Nan Zhang, Guoli Song, Hantao Wang

**Affiliations:** 1 State Key Laboratory of Cotton Bio-Breeding and Integrated Utilization, Institute of Cotton Research, Chinese Academy of Agricultural Sciences, Anyang 455000, China; 18167682847@163.com (M.W.); al07183361@163.com (L.A.); xkang_2010@163.com (X.F.); lujh212760@163.com (J.L.); hm651341@163.com (H.W.); 13721600131@163.com (Y.G.); zhang18799926748@163.com (N.Z.); 2 Guangdong Provincial Key Laboratory of Green and Intelligent Agricultural Production, College of Biological and Food Engineering, Guangdong University of Petrochemical Technology, Maoming 525000, China; cshen@gdupt.edu.cn; 3 National Key Laboratory of Cotton Bio-Breeding and Integrated Utilization, School of Life Sciences, Henan University, Kaifeng 475004, China

**Keywords:** upland cotton, *GhRNG1L*, drought tolerance, *GhDREB2B*

## Abstract

Drought stress is a major abiotic factor limiting cotton yield and quality. RING-type E3 ubiquitin ligases play central regulatory roles in plant stress responses; however, the functions and regulatory networks of this gene family in cotton drought response remain largely unclear. In this study, we characterized the RING-type E3 ubiquitin ligase gene *GhRNG1L* from upland cotton (*Gossypium hirsutum* L.) and demonstrated its role in enhancing drought tolerance in cotton, and we successfully identified its upstream transcription factor *GhDREB2B*. Functional assays showed that overexpression of *GhRNG1L* significantly increased catalase (CAT) activity and reduced hydrogen peroxide (H_2_O_2_) accumulation in *Arabidopsis*, thereby improving plant drought tolerance, whereas silencing *GhRNG1L* produced the opposite phenotype. Further molecular analysis confirmed that *GhDREB2B* directly binds to the promoter of *GhRNG1L* and activates its transcription. Collectively, our findings reveal, for the first time, the positive regulatory role of the *GhDREB2B-GhRNG1L* module in cotton drought response, providing a clear upstream regulatory target and theoretical basis for molecular breeding of drought-tolerant cotton.

## 1. Introduction

Cotton, as an indispensable source of natural fiber for the global textile industry, plays a vital role in global economic development [1]. However, in recent years, alongside the large-scale expansion of cotton cultivation, environmental issues such as exacerbated soil salinization, water scarcity, and extreme temperatures have become increasingly prominent [2,3]. Among the diverse abiotic stresses faced by cotton, drought stress is undoubtedly one of the most severe [4]. It not only inflicts substantial damage on the ecological environment but also significantly impairs cotton yield and quality, thereby weakening the sustainability of the cotton industry [2]. Therefore, screening elite drought-tolerant cultivars, elucidating the molecular mechanisms underlying drought tolerance, and enhancing drought resistance are of profound practical significance for the sustainable development of this crop.

Drought stress constrains plant growth and development, impairing morphogenesis, physiology, yield, and quality [5]. Water deficit suppresses cell division and elongation, causing stunted growth and leaf senescence [6], triggers stomatal closure that compromises CO_2_ assimilation [3], and damages photosynthetic structures [7]. Moreover, drought disrupts ROS homeostasis, leading to oxidative stress, membrane disintegration, and biomacromolecule degradation [8,9]. Collectively, these effects impair reproductive development and ultimately reduce yield and quality [10]. Plants have evolved sophisticated molecular strategies to counter drought stress [11]. Upon water deficit, both ABA-dependent and ABA-independent signaling pathways are rapidly activated [12,13], inducing drought-responsive transcription factors such as bZIP, MYB, NAC, and AP2/ERF family members [14,15]. These regulators control downstream genes involved in osmotic adjustment, antioxidative defense (SOD, POD, CAT), and membrane remodeling, thereby preserving cellular homeostasis. Among the AP2/ERF superfamily, DREB proteins play critical roles in drought tolerance [16]. Overexpression of *GmDREB1* or *EaDREB2* enhances drought and osmotic tolerance in transgenic wheat and sugarcane, respectively [17,18]. DREB proteins harbor a conserved AP2 domain that specifically binds DRE/CRT cis-elements in target gene promoters [18], modulating stress-responsive genes and ABA biosynthesis-related enzymes to reinforce drought resistance [19].

However, plant drought responses also depend on post-translational regulation [11]. Protein ubiquitination, a key post-translational modification (PTM) in eukaryotes, is essential for growth, cell cycle, and stress adaptation [20,21,22]. The ubiquitin–proteasome system (UPS) precisely controls protein abundance to reshape the proteome [21], conferring adaptive potential under abiotic stress. Ubiquitination is a cascade mediated by E1, E2, and E3 enzymes [23]. Plant E3 ligases are classified into HECT, RING, U-box, and Cullin-RING ligase complexes (CRLs) based on conserved domains and catalytic mechanisms [24,25], with the first three being single-subunit and CRLs multi-subunit [26]. Among the various E3 ligases, RING-type E3 ligases are the most abundant and play a central regulatory role in plant drought responses [27], with functions encompassing abscisic acid (ABA) signaling [28,29], stomatal movement regulation, reactive oxygen species (ROS) homeostasis maintenance, and cross-regulation among multiple signaling pathways [30]. In cotton, RING-type E3 ubiquitin ligases also play important roles [31]. For instance, silencing *GhSINAT5* in cotton impairs root development and reduces antioxidant enzyme activity, thereby significantly compromising plant drought and salt tolerance [32]. Although studies on *GhSINAT5* have demonstrated the positive effects of certain ligases under stress conditions, the biological functions of the vast majority of family members remain largely unknown. Therefore, the specific roles of RING-type E3 ligases in cotton drought tolerance warrant further investigation.

In this study, we cloned the *GhRNG1L* gene from *Gossypium hirsutum* L. Heterologous expression in *Arabidopsis* enhanced drought tolerance compared to the wild type (WT). Virus-induced gene silencing (VIGS) experiments in cotton showed that, compared with control plants, the silenced plants exhibited leaf wilting and water loss. Furthermore, we identified an upstream transcription factor, *GhDREB2B*, and Dual-Luciferase Assays confirmed that GhDREB2B interacts with the *GhRNG1L* promoter to activate its expression, thereby improving drought tolerance. Collectively, these results demonstrate that *GhRNG1L* plays a crucial regulatory role in drought resistance in cotton.

## 2. Results

### 2.1. Cloning and Sequence Analysis of GhRNG1L

**Figure 1:**
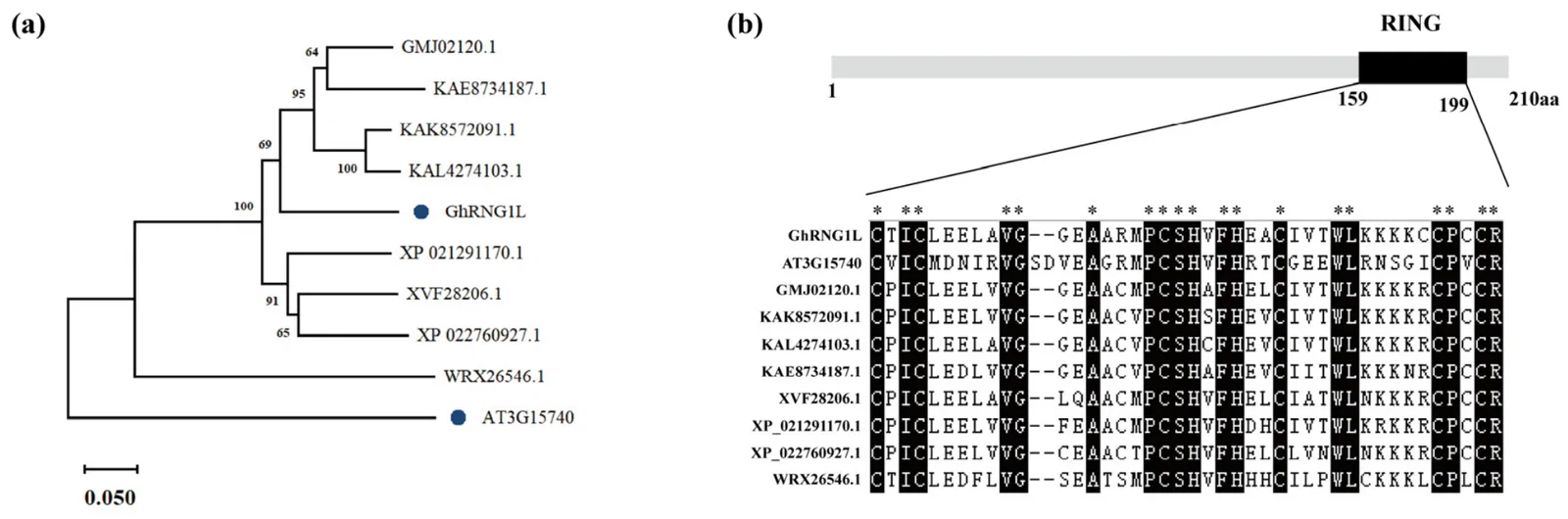
Phylogenetic tree and conserved domain analysis of the GhRNG1L protein. (**a**) Phylogenetic analysis of GhRNG1L and its orthologs from different species, including *Hibiscus trionum* (Ht), *Hibiscus sabdariffa* (Hsa), *Hibiscus cannabinus* (Hc), *Hibiscus syriacus* (Hsy), *Reevesia pubescens* (Rp), *Herrania umbratical* (Hu), *Durio zibethinus* (Dz), *Theobroma cacao* (Tc), and *Arabidopsis thaliana* (At). The phylogenetic tree was constructed using MEGA-X (version 12.1) software. (**b**) Schematic illustration of the structure of GhRNG1L and sequence alignment of the RING domain among GhRNG1L and its orthologs from different species. Asterisks indicate conserved cysteine and histidine residues.

To investigate the evolutionary relationships of *GhRNG1L* (*GH_D02G0591*) in upland cotton, 10 homologous protein sequences were selected from species of Malvales and other taxa to construct a phylogenetic tree (Figure 1a). The results showed that GhRNG1L clustered together with homologs from *Hibiscus trionum* (*GMJ02120.1*) and *Hibiscus syriacus* (*KAE8734187.1*), forming a clade with extremely high sequence conservation. Additionally, it was also placed within the same major clade as homologs from other Malvales species, including *Reevesia pubescens*, *Durio zibethinus*, *Theobroma cacao* and *Arabidopsis thaliana*, indicating that GhRNG1L is highly conserved during the diversification of Malvales species.

The GhRNG1L protein consists of 210 amino acids, with a molecular weight of 22.83 kDa, a theoretical isoelectric point (pI) of 4.33, and no signal peptide. A highly conserved RING finger domain is located at its C-terminus (amino acid region 159–199). Sequence alignment of the GhRNG1L amino acid domain with other sequences (Figure 1b) showed that GhRNG1L is highly identical to the other nine homologous proteins at multiple key positions. This region contains several completely conserved cysteine (Cys, C) and histidine (His, H) residues that together form a typical C3HC4-type RING motif. These results indicate that GhRNG1L is a member of the RING-type E3 ubiquitin ligase family and may exert corresponding biological functions.

### 2.2. Expression Pattern and Subcellular Localization of GhRNG1L

**Figure 2:**
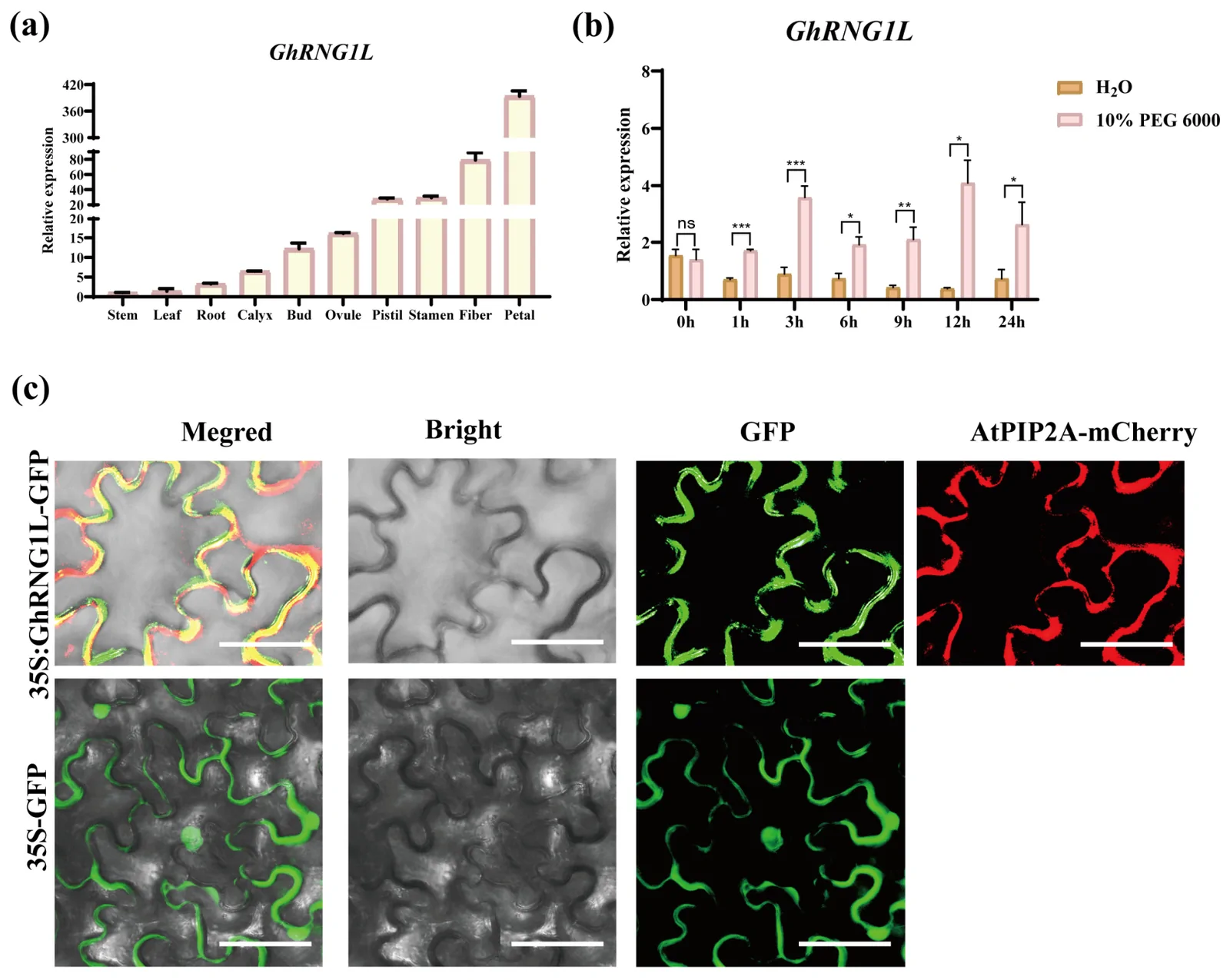
Tissue-specific expression, drought stress-responsive expression pattern, and subcellular localization of *GhRNG1L*. (**a**) Expression pattern of *GhRNG1L* in different tissues. (**b**) Expression pattern of *GhRNG1L* under normal conditions and 10% PEG 6000-simulated drought stress. (**c**) Subcellular localization of GhRNG1L. The green and red signals indicate GFP fluorescence and the plasma membrane marker AtPIP2A-mCherry, respectively, and the empty 35S-GFP vector control showing diffuse fluorescence. Bars = 50 μm. *, ** and *** indicate statistically significant differences at *p* < 0.05, *p* < 0.01, *p* < 0.001 and ns (not significant, *p* > 0.05) respectively (Student’s *t*-test).

The spatiotemporal expression pattern of gene is often closely related to its biological function. To preliminarily explore the potential function of *GhRNG1L*, qRT-PCR was used to examine its tissue-specific expression in upland cotton TM-1. The results showed that *GhRNG1L* was expressed in multiple tissues, with higher expression levels in petals, fibers and pistils, and lower expression levels in leaves, indicating tissue-specific expression (Figure 2a).

To further determine whether *GhRNG1L* is involved in the stress response of cotton, TM-1 plants were subjected to drought treatment, and the expression pattern of *GhRNG1L* under drought stress was examined. The results showed that the expression level of *GhRNG1L* in the control group (H_2_O treatment) remained at a low level throughout the treatment period. Under drought stress simulated by 10% PEG 6000, the expression level of this gene exhibited a fluctuating upward trend. It became significantly higher than that of the control group at 1 h after treatment, reached the first peak at 3 h, then declined at 6 h, but increased continuously from 9 h onward, reaching the highest value at 12 h (approximately 10-fold). These results indicate that *GhRNG1L* positively responds to drought stress, and its multi-peak fluctuating expression pattern suggests that this gene may be dynamically regulated at different stages of drought stress (Figure 2b).

Since *GhRNG1L* responds to drought stress, to further elucidate the molecular basis of its response to abiotic stress at the cellular level, we conducted a subcellular localization analysis of its encoded protein. A preliminary prediction of GhRNG1L subcellular localization using the online tool Cell-PLoc (http://www.csbio.sjtu.edu.cn/bioinf/Cell-PLoc/ (accessed on 15 April 2026)) indicated that GhRNG1L is localized to the plasma membrane. Subsequently, experimental validation was performed by constructing a *GhRNG1L*-GFP fusion vector, with the empty 35S-GFP vector serving as a negative control. Confocal laser scanning microscopy fluorescence imaging showed that the green fluorescence of *GhRNG1L*-GFP overlapped with the red fluorescence of the plasma membrane marker, whereas the 35S-GFP empty vector control exhibited diffuse green fluorescence throughout the entire cell. These results further confirm that GhRNG1L is localized to the plasma membrane (Figure 2c).

### 2.3. Overexpression of GhRNG1L Enhances Drought Tolerance in Transgenic Arabidopsis

**Figure 3:**
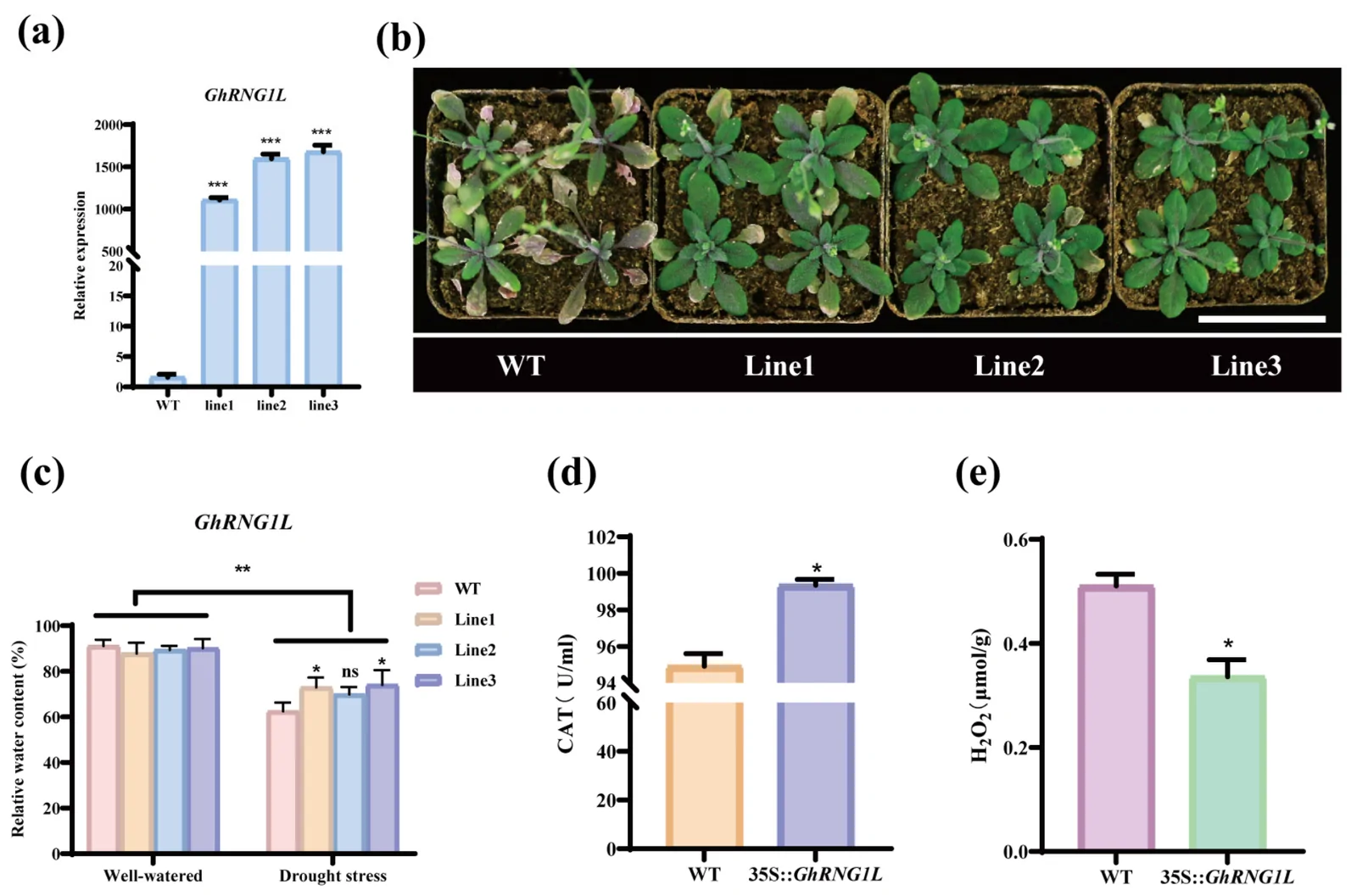
*GhRNG1L*-overexpressing *Arabidopsis* plants under drought treatment. (**a**) Relative expression levels of *GhRNG1L* in the leaves of transgenic *Arabidopsis* compared to WT. (**b**) Phenotypic comparison of transgenic and WT plants after 10 days of natural drought treatment initiated at four weeks of age. Bars = 4 cm. (**c**) Relative water content of WT and *GhRNG1L*-overexpressing *Arabidopsis* plants before and after drought treatment. (**d**) CAT activity in leaves of transgenic and WT after natural drought treatment. (**e**) H_2_O_2_ content in leaves of *GhRNG1L* overexpression lines and WT after natural drought treatment. Data are shown as means ± SD from three biological replicates (*n = 3*). *, ** and *** indicate statistically significant differences at *p* < 0.05, *p* < 0.01, *p* < 0.001 and ns (not significant, *p* > 0.05) respectively (Student’s *t*-test).

To investigate the functional role of *GhRNG1L* in drought stress, *GhRNG1L* was overexpressed in *Arabidopsis thaliana* to generate T_3_ transgenic lines. The expression levels of *GhRNG1L* in these overexpression lines were verified using qRT-PCR (Figure 3a). The results showed that the transcript levels in three independent overexpression lines were significantly higher than those in the WT plants (Figure 3b). Before drought treatment, the growth status of WT and transgenic lines was comparable under normal conditions (Figure S1), indicating that overexpression of *GhRNG1L* did not markedly affect normal growth of *Arabidopsis* plants. After being grown under uniform conditions, both transgenic and wild-type (WT) plants at four weeks of age were subjected to a 10-day natural drought treatment to observe their phenotypes and measure relevant physiological indices. The results showed that leaves of the WT plants exhibited wilting, chlorosis, and yellowing, whereas the transgenic lines still maintained green rosette leaves and showed overall better growth than the WT plants. Before drought treatment, there was no significant difference in relative water content between WT and overexpression lines; however, after drought treatment, the relative water content of overexpression lines was significantly higher than that of WT plants (Figure 3c). Consistently, the catalase (CAT) activity in the overexpression lines was significantly higher than that in the WT plants (Figure 3d), while the hydrogen peroxide (H_2_O_2_) content in the overexpression lines was lower than that in the WT plants (Figure 3e). Taken together, overexpression of *GhRNG1L* significantly enhanced drought resistance in *Arabidopsis*.

### 2.4. Silencing of GhRNG1L Reduces Drought Tolerance in Cotton

**Figure 4:**
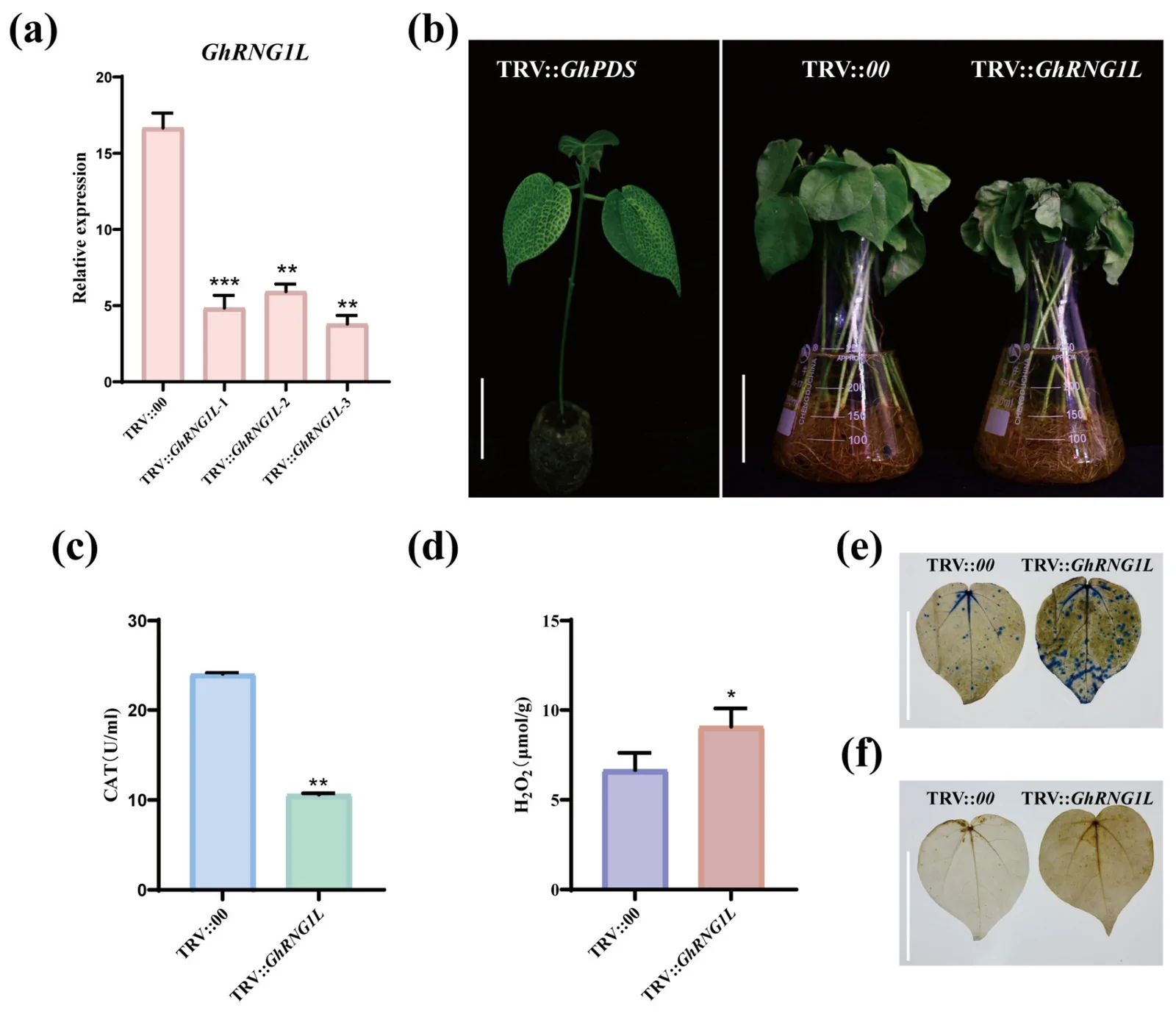
Silencing of *GhRNG1L* reduces drought tolerance in cotton. (**a**) Silencing efficiency of *GhRNG1L* in the leaves of TRV::*GhRNG1L* plants. (**b**) Albino phenotype of TRV::*GhPDS*; phenotypes of TRV::00 and TRV::*GhRNG1L* plants after 12 h of 10% PEG6000 treatment. Bars = 4 cm. (**c**) CAT activity in leaves of TRV::00 and TRV::*GhRNG1L* plants under drought treatment. (**d**) H_2_O_2_ content in leaves of TRV::00 and TRV::*GhRNG1L* plants under drought treatment. (**e**) Trypan blue staining of leaves of TRV::00 and TRV::*GhRNG1L* plants under drought treatment. Bars = 5 cm. (**f**) DAB staining of leaves of TRV::00 and TRV::*GhRNG1L* plants under drought treatment. Bars = 5 cm. Data are shown as means ± SD from three biological replicates (*n = 3*). *, ** and *** indicate statistically significant differences at *p* < 0.05, *p* < 0.01, *p* < 0.001 and ns (not significant, *p* > 0.05) respectively (Student’s *t*-test).

To further verify the role of *GhRNG1L* under drought stress, virus-induced gene silencing (VIGS) was used to reduce the expression level of *GhRNG1L* in cotton. VIGS cotton plants with *GhRNG1L* silencing (TRV::*GhRNG1L*), negative control plants (TRV::00), and positive control plants (TRV::*GhPDS*) were generated. When the TRV::*GhPDS* plants exhibited an albino phenotype, it indicated that the VIGS system functioned properly. Total RNA was extracted from the leaves to determine the silencing efficiency. Compared with the negative control, the expression of *GhRNG1L* in TRV::*GhRNG1L* plants was significantly downregulated, confirming that the gene was efficiently silenced in TM-1 (Figure 4a). The silenced plants and negative control plants were then subjected to drought treatment using 10% PEG6000 for 12 h, and changes in physiological indicators under drought stress were examined.

Before drought stress, the negative control and silenced plants showed comparable growth status under normal conditions (Figure S2), indicating that silencing *GhRNG1L* did not markedly affect normal growth of cotton plants. Upon drought stress, the results showed that compared with the negative control plants, the silenced plants exhibited more severe wilting (Figure 4b). The CAT activity in the leaves of the silenced plants was significantly lower than that in the control plants (Figure 4c), while the H_2_O_2_ content was extremely significantly higher than that in the control plants (Figure 4d). Additionally, trypan blue and DAB staining were performed on both silenced and control plants. Trypan blue staining revealed that the leaves of silenced plants showed a large area of dark blue coloration, whereas the control leaves exhibited only a small amount of light blue staining (Figure 4e). DAB staining revealed that the brown precipitate in the leaves of silenced plants was significantly darker, whereas the brown coloration in the control leaves was lighter (Figure 4f). The staining results were further quantified using image analysis software, and this confirmed the same trend (Figure S3). Thus, silencing *GhRNG1L* exacerbated cell death and H_2_O_2_ accumulation under drought stress, indicating that *GhRNG1L* positively regulates drought response in upland cotton.

### 2.5. Analysis of GhRNG1L Promoter Elements and Prediction of Upstream Regulatory Factors

**Figure 5:**
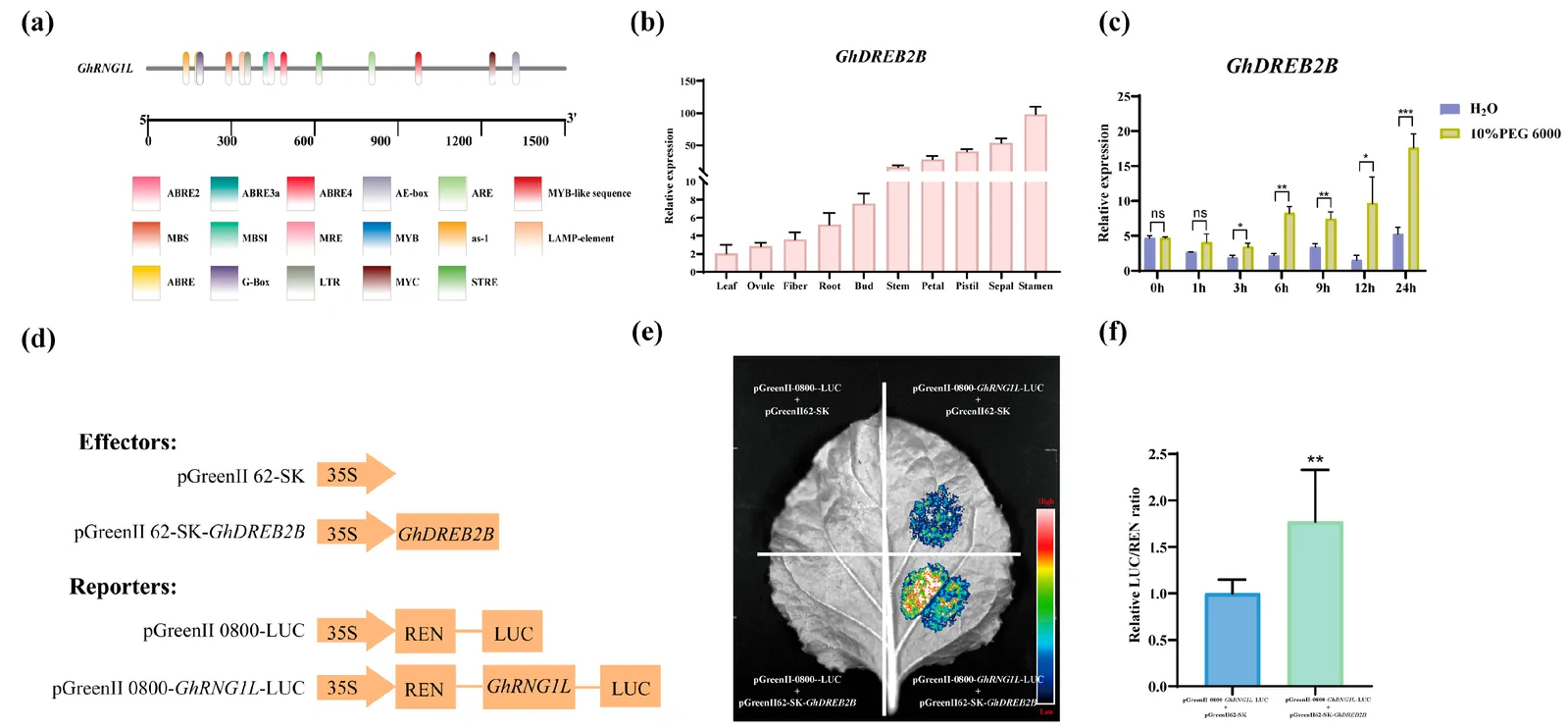
Expression patterns of *GhDREB2B* and its transcriptional regulation of the *GhRNG1L* promoter. (**a**) Analysis of *GhRNG1L* promoter elements. (**b**) Expression pattern of *GhDREB2B* in different tissues of cotton TM-1. (**c**) Expression pattern of *GhDREB2B* under normal conditions and 10% PEG 6000-simulated drought stress. (**d**) Schematic diagram of the vector construction for the Dual-Luciferase reporter assay. (**e**) Transactivation analysis using a Dual-Luciferase system showing that GhDREB2B positively regulates *GhRNG1L* promoter activity. (**f**) Detection of relative luciferase activity of GhDREB2B and *GhRNG1L* promoter. Data are shown as means ± SD from three biological replicates (*n = 3*). *, ** and *** indicate statistically significant differences at *p* < 0.05, *p* < 0.01, *p* < 0.001 and ns (not significant, *p* > 0.05) respectively (Student’s *t*-test).

To further investigate the regulatory mechanism and potential functions of *GhRNG1L* in stress tolerance, the 1475-bp promoter sequence upstream of the *GhRNG1L* start codon (ATG) was extracted, and analyzed for cis-acting elements in the promoter sequence using the PlantCARE website (https://bioinformatics.psb.ugent.be/webtools/plantcare/html/ (accessed on 17 March 2026)). The results demonstrated that this promoter region contained basal and light-responsive elements, such as G-Box and MRE, and was significantly enriched with cis-acting elements closely associated with drought. Specifically, these stress-responsive elements included a drought-inducible element (MBS), a stress-responsive element (STRE), and an anaerobic-responsive element (ARE). The hormone-responsive elements contained a large number of ABA (abscisic acid)-responsive elements, such as ABRE2, ABRE3a, and ABRE4 (Figure 5a). The dense distribution of these regulatory elements suggests that the *GhRNG1L* gene is likely involved in the response to drought stress.

Given that the promoter region is highly enriched with specific drought and hormone-responsive elements, we hypothesized that the expression of *GhRNG1L* is precisely regulated by upstream transcription factors. Accordingly, we predicted and screened upstream regulators of *GhRNG1L* using the PlantTFDB website (https://plantregmap.gao-lab.org/binding_site_prediction.php (accessed on 8 March 2026)). Considering the central role of the Dehydration-Responsive Element-Binding (DREB) family in plant drought and ABA signal transduction pathways, along with the evidence that *GhDREB2B* can regulate drought stress responses and is significantly up-regulated in cotton under drought stress [33], we identified the transcription factor *GhDREB2B* (*GH_D09G1791*) as a candidate upstream regulator. The full-length cDNA of *GhDREB2B* was cloned from the standard genetic material upland cotton TM-1. The open reading frame (ORF) of this gene is 1530 bp in length and encodes a protein of 509 amino acids (aa). The GhDREB2B protein has a molecular weight of 57.24 kDa, a theoretical isoelectric point (pI) of 4.7989, and contains no signal peptide.

### 2.6. Expression Pattern of GhDREB2B

To investigate the tissue-specific expression pattern of *GhDREB2B*, qRT-PCR was used to examine its expression levels in different tissues. The results showed that *GhDREB2B* was expressed in various tissues, with relatively high expression in stamen, sepal, and pistil, and relatively low expression in leaf, indicating a clear tissue-specific expression pattern (Figure 5b).

To further clarify the response characteristics of *GhDREB2B* to drought stress, cotton TM-1 plants were subjected to drought treatment, and the expression dynamics of this gene were examined by qRT-PCR at different time points (0, 1, 3, 6, 9, 12, and 24 h). The results showed that in the control group (treated with H_2_O), the expression level of *GhDREB2B* remained low at all time points. In contrast, under drought stress simulated by 10% PEG 6000, the expression of *GhDREB2B* exhibited a significant and sustained upward trend, becoming significantly higher than that of the control group at 3 h after treatment, and continuing to increase over time, peaking at 12 h (approximately 6-fold). These results indicate that *GhDREB2B* positively responds to drought stress (Figure 5c).

### 2.7. GhDREB2B Positively Regulates the Expression of GhRNG1L

To investigate the molecular mechanism by which *GhRNG1L* responds to drought stress, potential upstream regulators were analyzed. A Dual-Luciferase Assay (DLR) was performed to verify the interaction between GhDREB2B and the *GhRNG1L* promoter. A 1475-bp fragment of the *GhRNG1L* promoter was inserted into the reporter vector, while *GhDREB2B* was cloned into the effector vector (Figure 5d). After co-expression of the 35S::*GhDREB2B* effector construct and the *GhRNG1L* promoter-driven reporter construct in *Nicotiana benthamiana* leaves, *GhRNG1L* expression was strongly activated by GhDREB2B (Figure 5e,f). This indicates that GhDREB2B enhances the activity of the *GhRNG1L* promoter, thereby positively regulating its expression in vivo, and that GhDREB2B may function as a transcriptional regulator of *GhRNG1L*.

## 3. Discussion

In this study, bioinformatics analysis revealed that the GhRNG1L protein contains a typical RING domain and is predicted to be a RING-type E3 ubiquitin ligase. Sequence alignment and conserved motif analysis further showed that GhRNG1L shares high similarity with known classical RING-type E3 ubiquitin ligases, suggesting that it may be a member of this family and possess corresponding molecular functions. However, these inferences are based solely on sequence-level predictions, and its actual ubiquitin ligase activity or function remains to be verified through in vitro ubiquitination assays [34]. Confirmation of this enzymatic activity would provide a necessary prerequisite for investigating the transcriptional regulation of *GhRNG1L* by the upstream transcription factor GhDREB2B. On this basis, future efforts employing co-immunoprecipitation coupled with mass spectrometry (IP-MS) or yeast two-hybrid (Y2H) screening to identify interacting proteins/substrates of GhRNG1L will help to further elucidate the molecular network through which this ligase regulates drought tolerance in cotton.

The spatiotemporal expression characteristics of a gene are often closely related to its biological function. In this study, a RING-type E3 ubiquitin ligase gene, *GhRNG1L*, was cloned from *Gossypium hirsutum* L. Functional analysis showed that under drought stress simulated by 10% PEG 6000, *GhRNG1L* exhibited a fluctuating upward expression pattern, indicating that it positively responds to drought and plays regulatory roles at different stages. Furthermore, under drought stress, the phenotype and physiological parameters of transgenic *Arabidopsis* overexpressing *GhRNG1L* showed enhanced drought tolerance compared with the wild type, whereas silencing *GhRNG1L* in upland cotton led to opposite effects. Meanwhile, tissue-specific qRT-PCR analysis revealed that *GhRNG1L* was expressed in various cotton tissues but exhibited obvious tissue specificity, with significantly higher expression levels in floral organs such as petals, fibers, and pistils than in other tissues such as leaves. The relatively high expression of *GhRNG1L* in floral organs suggests a potential role in these tissues, which warrants further investigation. Currently, relevant studies have shown that E3 ubiquitin ligases are key factors in the regulation of flowering time in plants. They precisely regulate flowering by mediating the ubiquitination and degradation of core flowering regulatory proteins. For example, the E3 ubiquitin ligase *AtHOS1* directly ubiquitinates and degrades the key flowering activator CONSTANS (CO) protein under light, forming a temporal division of labor with *AtCOP1*, which acts at night, thereby ensuring the accuracy of photoperiodic flowering by preventing the accumulation of CO in the morning [35]. The U-box E3 ubiquitin ligase AtPUB13 forms a complex with AtBON1 protein to coordinately negatively regulate flowering; loss of function of either component downregulates the expression of *FLC*, leading to early flowering [36].

Under drought stress, plants accumulate large amounts of ROS, leading to oxidative stress and programmed cell death [37,38]. To cope with oxidative damage, plants have evolved an ROS-scavenging system centered on CAT, SOD, and POD [39], among which CAT, as a rate-limiting enzyme, catalyzes the decomposition of highly toxic H_2_O_2_ into harmless H_2_O and O_2_, thereby preventing ·OH production and maintaining ROS signaling homeostasis. Recent studies have shown that the *Arabidopsis* RING-type E3 ligase *CIRP1* directly binds to *CAT2* and *CAT3* and mediates their ubiquitination and degradation; accordingly, the *cirp1* mutant exhibits significantly elevated CAT activity and reduced H_2_O_2_ accumulation, conferring enhanced tolerance to drought and oxidative stress [40]. Beyond directly targeting antioxidant enzymes such as CAT, E3 ligases more broadly regulate ROS homeostasis through the ubiquitination and degradation of nuclear transcription factors. For instance, the *Arabidopsis* RING-type E3 ligases *MIEL1* and *LbRZF1* mediate the ubiquitination and degradation of *MYB30* and *MYB113*, respectively, thereby relieving the transcriptional repression imposed by these transcription factors on downstream target genes and modulating the expression networks of stress-responsive and ROS-related genes [41,42]. Collectively, these findings indicate that RING-type E3 ubiquitin ligases participate in ROS homeostasis regulation by targeting key proteins at multiple levels. Recent single-cell transcriptomic studies have revealed cell type-specific transcriptional programs in response to abiotic stress [43], suggesting that *GhRNG1L*-mediated ROS regulation may exhibit tissue- or cell-type specificity. Based on these observations, we speculate that *GhRNG1L* may similarly participate in the regulation of ROS homeostasis in cotton through the aforementioned direct or indirect pathways.

Plant drought tolerance is coordinately regulated by multiple hormonal signals, among which abscisic acid (ABA) and auxin are two core phytohormones. Auxin enhances plant adaptability to environmental stress by modulating stress signaling pathways and interacting with other hormones [44]. Meanwhile, ABA coordinates plant adaptive responses to water deficit through multiple pathways, including regulation of stomatal movement, root architecture, and stress-responsive gene expression [14,15]. Although DREB transcription factors traditionally function in ABA-independent pathways, recent studies show they also interweave with ABA signaling [45]. Given that the upstream regulator GhDREB2B links various stress signals, its downstream target, *GhRNG1L*, is speculated to participate in the ABA signaling network. Supporting this hypothesis, promoter analysis revealed abundant ABA-responsive cis-acting elements in *GhRNG1L*. Furthermore, GhRNG1L localizes to the plasma membrane, which is often the critical hub for ABA perception and transmembrane transport. This localization resembles that of *AtCHYR1*, a plasma membrane-localized RING E3 ligase known to mediate guard cell ROS production and induce stomatal closure under stress [46]. Based on the above evidence, we propose a speculative model to illustrate the possible mechanism by which *GhRNG1L* enhances drought tolerance in cotton through the regulation of ABA signaling. *GhRNG1L* may dynamically modulate ABA signaling intensity via a negative feedback loop. Drought stress induces endogenous ABA accumulation, which activates *GhRNG1L* transcription; subsequently, the GhRNG1L protein, through its E3 ligase activity, may target positive regulators of ABA signaling and mediate their degradation, thereby attenuating ABA signal output and reducing its own transcriptional level. As GhRNG1L protein levels decline, the positive regulators are restored, allowing cells to regain responsiveness to ABA. This negative feedback mechanism not only prevents damage caused by signal overactivation but also sustains the plant’s flexible adaptation to prolonged stress.

## 4. Materials and Methods

### 4.1. Plant Materials and Culture Conditions

In this study, the plant materials used were *Arabidopsis thaliana* Columbia-0 wild type (WT), *Nicotiana benthamiana*, and *Gossypium hirsutum* L. TM-1. All plants were sown in a mixed substrate consisting of nutrient soil (Pindstrup Mosebrug A/S, Ryomgård, Denmark) and vermiculite at a ratio of 2:1 (*v*/*v*). Before sowing, Seeds of TM-1 plants were soaked overnight until germination and *Arabidopsis* seeds were treated at 4 °C for 2–3 days before sowing. *N. benthamiana* seeds were sown directly without pretreatment. All plants were grown under uniform conditions: 25 °C day/22 °C night, a 16 h light/8 h dark photoperiod, and 60–70% relative humidity.

When the cotton VIGS plants reached the three-leaf-one-heart stage, they were removed from the solid substrate, and the roots were gently rinsed with deionized water to remove residual substrate, then transferred to a 10% PEG6000 (Solarbio, P8250, Beijing, China) solution for 12 h. When the *Arabidopsis* plants grew to four weeks of age, they were treated with natural drought stress by withholding water for 10 days. After treatment, leaves were collected from the plants, immediately frozen in liquid nitrogen, and stored at −80 °C for later use. Additionally, upland cotton TM-1 plants at the three-leaf-one-heart stage were subjected to 10% PEG 6000 or water treatment, and samples were harvested at 0, 1, 3, 6, 9, 12, and 24 h post-treatment, and stored in the same manner as previously described.

### 4.2. Phylogenetic Tree Construction and Multiple Sequence Alignment

The physicochemical properties of GhRNG1L and GhDREB2B proteins were predicted using the online ProtParam tool on the ExPASy server (http://web.expasy.org/protparam/ (accessed on 24 January 2026)), with a focus on core parameters including molecular weight (MW) and theoretical isoelectric point (pI) [47]. Meanwhile, the phylogenetic tree was constructed using MEGA-X (version 12.1) software.

### 4.3. Subcellular Localization Assay

The 35S-2300-GFP vector was linearized by digestion with XbaI and BamHI. The coding sequence (CDS) of *GhRNG1L* was amplified from upland cotton TM-1 and inserted into the linearized vector by homologous recombination to generate the fusion expression vector 35S-*GhRNG1L*-GFP. The empty 35S-2300-GFP vector was used as a control. The resulting recombinant plasmids and the empty vector were individually introduced into leaves of 4- to 6-week-old *Nicotiana benthamiana* plants by *Agrobacterium*-mediated infiltration. After infiltration, the leaves were kept in darkness at room temperature for 24 h and then incubated under low-light conditions for an additional 6–12 h. The subcellular localization of the GhRNG1L protein was then examined under a fluorescence microscope, with AtPIP2A-mCherry serving as a membrane marker [48]. Primer sequences are listed in Table S1.

### 4.4. Quantitative Real-Time PCR Analysis

RNA was extracted from the leaves using the FastPure Universal Plant Total RNA Isolation Kit (Vazyme, RC411-01, Nanjing, China). Reverse transcription was performed with HiScript II qRT SuperMix containing gDNA wiper (Vazyme, R223-01, Nanjing, China). Quantitative real-time PCR (qRT-PCR) was carried out on an ABI 7500 platform (Applied Biosystems, Waltham, MA, USA) employing the UltraSYBR One Step qRT-PCR Kit (Cwbio, CW0659S, Taizhou, China). The cotton *GhHIS3* (*AF024716*) [49] and the *Arabidopsis UBQ10* (*AT4G05320*) gene were used as internal references for cotton and *Arabidopsis* samples, respectively, and relative expression levels were calculated using the 2^−ΔΔCt^ method. Data are presented as the mean ± standard deviation of three biological replicates [50]. The same kits were also used for expression analysis of T_3_ transgenic *Arabidopsis* lines and for verification of VIGS efficiency. All primer sequences are listed in Table S1.

### 4.5. Agrobacterium-Mediated Heterologous Expression in Arabidopsis

The *GhRNG1L* gene fragment was inserted into the pBI121 vector, which had been linearized via double digestion with XbaI and SacI. The resulting construct, pBI121-*GhRNG1L*, was transformed into *Agrobacterium tumefaciens* strain GV3101. Genetic transformation of wild-type *Arabidopsis thaliana* (Col-0) was performed using the standard floral dip method. *Agrobacterium* cells were collected and resuspended in transformation buffer (5% sucrose, 200 μM acetosyringone, and 20 μL Silwet L-77) to an OD_600_ of 0.8–1.0. Three-week-old *Arabidopsis* plants were then transformed by floral dip, kept in darkness under high humidity for 24 h, and subsequently returned to normal greenhouse conditions. T_0_ seeds were collected and screened on 1/2 MS medium supplemented with kanamycin. Surviving T_1_ seedlings were transplanted into soil for further molecular identification and expression analysis. Finally, homozygous T_3_ transgenic lines were established and utilized for subsequent experiments. All primer sequences are listed in Table S1.

### 4.6. Virus-Induced Gene Silencing in Cotton

Virus-induced gene silencing (VIGS) exploits the endogenous RNA-mediated antiviral defense pathway in plants. This technique utilizes the Tobacco rattle virus (TRV) vector system, which consists of TRV1 (pYL192) and TRV2 (pYL156). A 300-bp *GhRNG1L* silencing fragment was designed using the online SGN VIGS Tool (https://vigs.solgenomics.net/). The designed *GhRNG1L*-specific fragment was cloned into the TRV2 (pYL156) vector to construct the pYL156-*GhRNG1L* vector. The resulting recombinant plasmid, the empty vector (TRV::00), and the positive control (TRV::*GhPDS*) were individually co-transformed with the helper vector TRV1 (pYL192) into *Agrobacterium tumefaciens* strain LBA4404. The bacterial cells were collected and resuspended in infiltration buffer (10 mM MgCl_2_, 10 mM MES, 200 μM acetosyringone) to an OD_600_ of 1.5. Equal amounts of TRV::00, TRV::*GhRNG1L*, TRV::*GhPDS*, and TRV1 (pYL192) were mixed and allowed to stand at room temperature in the dark for 3 h. Then, the mixture was injected into the cotyledons of cotton plants using a needleless syringe. Once the TRV::*GhPDS* plants exhibited the typical albino phenotype, RNA was extracted from each group of plants, and the silencing efficiency of the target gene was detected by qRT-PCR. Successfully silenced plants were used for subsequent experiments. All primer sequences are listed in Table S1.

### 4.7. Histochemical Staining and Biochemical Assays

To evaluate the extent of damage and physiological responses of plants under drought stress, cell damage assessment and related indicator measurements were performed in this study. Trypan blue and 3,3′-diaminobenzidine (DAB) staining were used to assess the degree of cell damage in cotton [49]. Leaves from drought-stressed VIGS plants were immersed in Trypan blue staining solution, boiled in a water bath for 2 min, and then destained with chloral hydrate (2.5 g/mL). Meanwhile, DAB staining was conducted on drought-stressed leaves using a DAB detection kit (the Nanjing Institute of Bioengineering) with the working solution prepared strictly according to the manufacturer’s instructions, followed by destaining with ethanol. After drought treatment, the H_2_O_2_ content and CAT activity in leaves of *Arabidopsis* overexpression lines and cotton VIGS plants were measured using the corresponding kits (Solarbio, Beijing, China) strictly following the manufacturer’s instructions; three biological replicates were performed for each experiment. Meanwhile, the relative water content (RWC) of *Arabidopsis* was also measured. Leaf samples from overexpression lines and wild-type controls were collected before and after drought treatment. Fresh weight (FW) was immediately determined upon excision. The samples were then submerged in deionized water and kept in darkness at 4 °C for 24 h to allow full hydration, after which turgid weight (TW) was recorded. Subsequently, the tissues were oven-dried at 80 °C for 48 h to obtain dry weight (DW). Relative water content was calculated using the following formula: RWC (%) = [(FW − DW)/(TW − DW)] × 100. Three biological replicates were performed for each line [51].

### 4.8. Dual-Luciferase Reporter Assay

The coding sequence (CDS) of the upstream transcription factor *GhDREB2B* was cloned into the effector vector pGreenII-SK-LUC, and the promoter of the target gene *GhRNG1L* (1475 bp) was cloned into the reporter vector pGreenII-0800-LUC. The recombinant plasmids were individually transformed into *Agrobacterium tumefaciens* GV3101, and the resulting *Agrobacterium* suspensions were mixed and co-infiltrated into leaves of *Nicotiana benthamiana*. After 48 h, luciferase activity was detected using the Tanon 5200 imaging system. All primer sequences are listed in Table S1.

## 5. Conclusions

In this study, establishes *GhRNG1L* as a positive regulator of drought tolerance in cotton. The key advance is the identification of a *GhDREB2B*–*GhRNG1L* transcriptional module in which the drought-responsive transcription factor GhDREB2B directly activates *GhRNG1L*; *GhRNG1L* in turn reinforces the antioxidant defense system, accelerates H_2_O_2_ scavenging, and mitigates oxidative damage. This module mechanistically links drought signaling to ROS homeostasis and offers a promising candidate for molecular breeding of drought-resistant cotton.

Nevertheless, several limitations should be acknowledged. Additionally, quantitative soil moisture data were not recorded during drought treatments, which limits the precision of stress intensity characterization. The direct ubiquitination substrates of GhRNG1L remain unidentified, and the contribution of its E3 ligase activity to drought tolerance has not been fully resolved at the molecular level. In addition, the current functional validation has been performed mainly through heterologous overexpression in *Arabidopsis* and VIGS-mediated transient silencing in cotton, and the breeding value of *GhRNG1L* remains to be verified in stable transgenic cotton lines and under field conditions.

## Data Availability

The original contributions presented in this study are included in the article/Supplementary Material. Further inquiries can be directed to the corresponding authors.

## Supplementary Materials

The following supporting information can be downloaded at: https://www.mdpi.com/article/10.3390/plants15172636/s1, Table S1. Primers used in this study. Table S2. Gene sequences used in the article. Figure S1. Growth status of WT and transgenic lines before drought treatment. Figure S2. Growth status of negative control and silenced plants before drought treatment. Figure S3. Image-based quantification of trypan blue and DAB staining.
