# Supplementary material for: Functional Analysis of GhRNG1L Reveals Its Positive Role in Drought Tolerance Mediated by GhDREB2B in Cotton
Source: Plants (Basel). 2026 Aug 28;15(17):2636. doi: 10.3390/plants15172636 (PMC13567090; doi:10.3390/plants15172636)
Supplement: Supplementary file 1 [file plants-15-02636-s001.zip › Figures S1-S3.pdf]

(a)

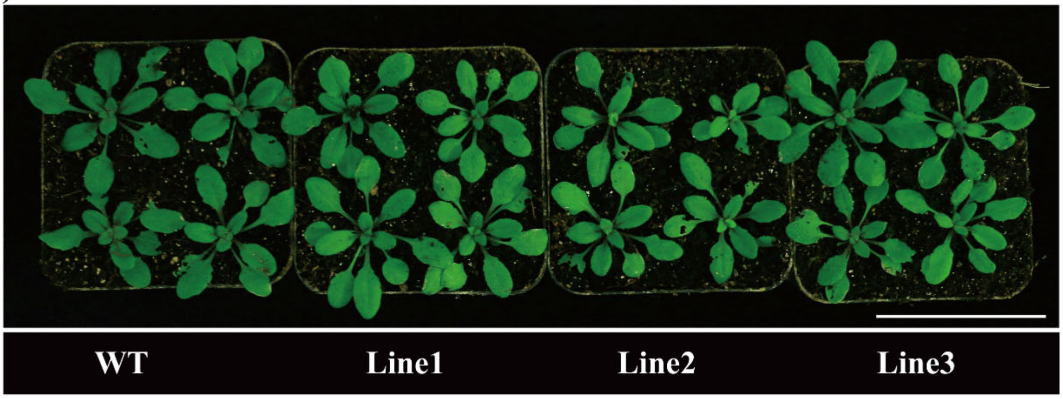

Figure S1 Growth status of WT and transgenic lines before drought treatment. Bars=4cm.

(b)

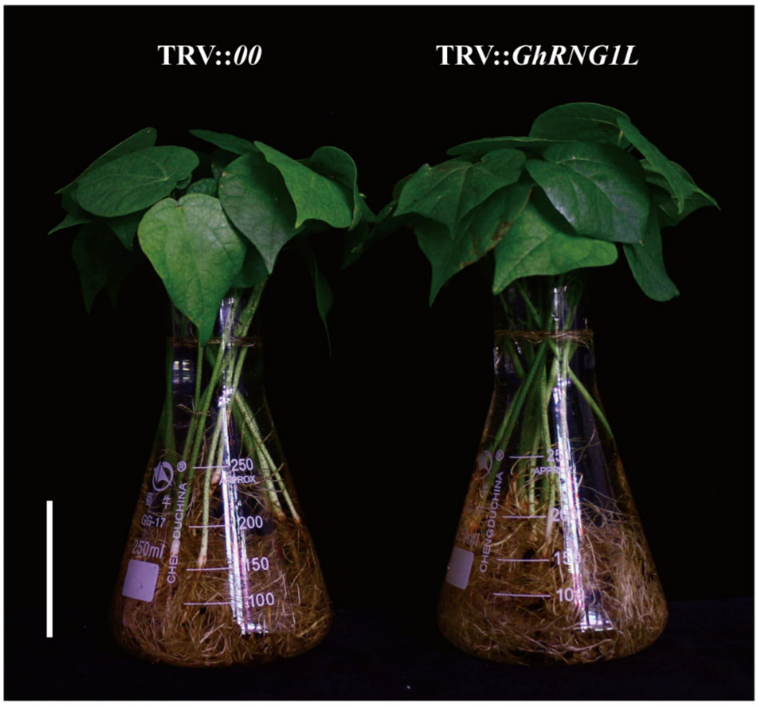

Figure S2 Growth status of negative control and silenced plants before drought treatment. Bars=4cm.

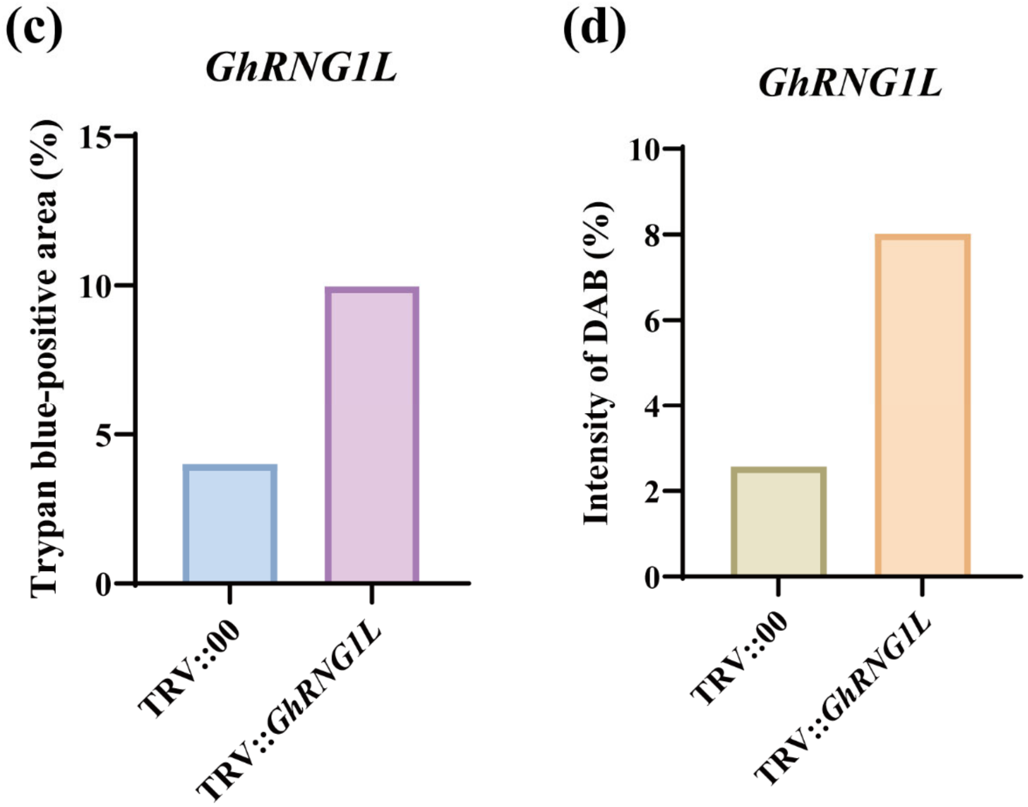

Figure S3 Image-based quantification of trypan blue and DAB staining.
